# Thiamine Deficiency Neuropathy in a Patient with Malnutrition due to Melancholic Depression

**DOI:** 10.1155/2024/1797983

**Published:** 2024-03-08

**Authors:** Rihene Melki, Rim Ben Soussia, Houcem Elomma Mrabet, Walid Bouali, Lazhar Zarrouk

**Affiliations:** ^1^Psychiatry Department, Taher Sfar University Hospital-Mahdia, Monastir University, Ksar Hallal, Monastir, Tunisia; ^2^Internal Medicine and Endocrinology Department, Taher Sfar University Hospital-Mahdia, Monastir University, Ksar Hallal, Monastir, Tunisia

## Abstract

**Introduction:**

Melancholic depression is a daily clinical reality in psychiatry. It is a therapeutic emergency that can jeopardize life if not promptly and adequately treated. Apart from its high suicidal risk, complications related to the under-nourishment state are to be feared. *Case Presentation*. A 36-year-old woman was admitted with depressive symptoms, significant weight loss, and total functional impotence. Laboratory investigations revealed severe thiamine (vitamin B1) deficiency. An electromyography confirmed a sensory axonal neuropathy involving all four extremities suggesting a deficiency origin. *Discussion*. Vitamin and mineral deficiencies have been described in patients with malnutrition resulting from psychiatric illness (anorexia nervosa, eating disorders, severe depression, etc.). Thiamine is an essential cofactor in several biochemical pathways. Its deficiency can lead to neuropsychiatric morbidity.

**Conclusion:**

In our case, the rapid weight loss facilitated a cascade of complications related to nutritional deficiencies. Based on our clinical observations and the literature, thiamine deficiency should be considered in the presence of malnutrition and vulnerability, both on an organic and psychiatric level.

## 1. Introduction

Depression is a common and serious public health concern [1]. According to the World Health Organization, approximately 4.4% of the global population was suffering from depression in 2015 [2].

In the DSM-5 classification [3], “melancholic features” represent a specifier of a major depressive episode. Psychomotor retardation and lack of mood reactivity are the most indicative clinical features of melancholic depression. Other symptoms include psychomotor agitation, early awakening, and, notably, loss of appetite and weight [4]. In the elderly, unexplained weight loss can be a prominent feature of melancholic depression [5].

A depressive episode can then lead to significant weight loss or even a state of undernutrition. Malnutrition or undernutrition is defined as “an imbalance in nutritional status” and is associated with increased morbidity and mortality [6, 7]. While malnutrition has become relatively rare, its diagnosis should raise suspicion of nutriment and vitamin deficiencies.

This case focuses on an unexpected neurological complication that arises from a melancholic state associated with nutritional deficiency.

## 2. Case Presentation

Mrs. H.S., a 36-year-old woman, presented with apathy and food refusal. The patient is described by her family as highly emotional and sensitive. The episode started 2 months prior, with rapidly progressive tendencies towards isolation, irritability, and increased religious concerns. The symptoms progressed into statements of hopelessness and self-depreciation, along with a refusal to eat. As the symptoms progressed, the family noticed a marked weight loss of 15 kg per month (15% of initial weight) as well as episodes of vomiting resistant to symptomatic treatment. The initial clinical evaluation revealed signs of extracellular dehydration, a BMI of 18 kg/m^2^, and a total functional impotence. The psychiatric examination revealed an alert, yet apathetic patient who remained in bed, had difficulty with social interactions, emotional numbness, irritability, and anxiety. She expressed delusional thoughts of hopelessness, punishment, and suicidal ideation.

The patient was admitted to a psychiatric department, and upon the reintroduction of oral nutrition, she reported abdominal pain, diarrhoea, tachypnoea, along with low oxygen levels. Biochemical analysis showed elevated blood sugar levels, low potassium, phosphate, and magnesium levels. Inappropriate refeeding syndrome (IRS) was diagnosed, and the patient was transferred to an intensive care unit for 4 days, where she received parenteral nutrition and vitamin supplementation (Vit B1, Vit B9, Vit D, and Vit E). The patient was then readmitted to the psychiatry department. The diagnosis of major depression with psychotic and melancholic features was made. The patient started on Venlafaxine at a dose of 150 mg/day in combination with Olanzapine.

Within 3 weeks of treatment, the patient became more responsive and less irritable. The delusional ideas disappeared, but total functional impotence persisted. The neurological examination was challenging; the patient was bedridden with flaccid tetraparesis, predominantly in the lower limbs. In this context, metabolic neuropathy due to vitamin deficiency was highly suspected. A thiamine dosage conducted during the patient's stay in the intensive care unit later revelled decreased levels at 36 nmol/L (normal range: (70–180) nmol/L). The patient was put on intravenous Vit B1 300 mg three times a day for 7 days. She was then switched on 100 mg of Vit B1 orally per day. Electromyography (EMG) of all four limbs showed severe axonal polyneuropathy, predominantly affecting the nerves of the lower limbs (Table 1). Brain and spinal cord MRI showed no abnormalities. The patient presented with acute peripheral neuropathy, probably due to malnutrition in the context of melancholic depression. The patient was maintained on antidepressant treatment and oral polyvitamin supplementation, along with multidisciplinary follow-up (physical medicine, neurology, psychiatry). Unfortunately, neurological deficits remain unrelieved.

## 3. Discussion

### 3.1. Thiamine Deficiency in the Context of Malnutrition

**Figure 1:**
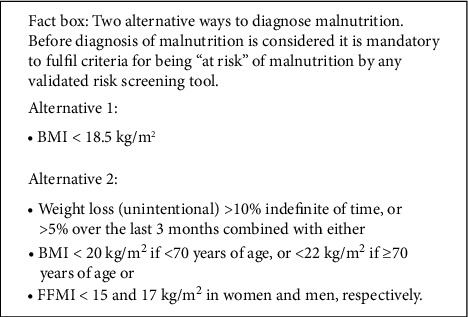
Diagnostic criteria for malnutrition according to the European Society of Clinical Nutrition and Metabolism (ESPEN) [10].

Malnutrition is an under-diagnosed, poorly documented phenomenon that negatively impact on the duration and quality of life [8, 9]. There are still no universal definitions for the terms undernutrition or malnutrition. In a recent consensus report in Clinical Nutrition, the undernourished category of malnutrition was proposed to be defined and diagnosed on the basis of a low BMI or unintentional weight loss combined with low BMI or fat-free mass index (fat-free mass corrected for body size: FFMI) with certain cut-off points. Diagnostic criteria for malnutrition according to The European Society of Clinical Nutrition and Metabolism (ESPEN) are summarised in Figure 1 [10]. In our patient, weight loss exceeded 15% of body weight, and her BMI was below 18.5 kg/m^2^. This disordered nutritional state led to diminished physical and mental function and impaired clinical outcomes.

Deficiencies in vitamins and minerals are described in patients with malnutrition resulting from psychiatric illness (anorexia nervosa, eating disorders, severe depression, etc.). In our case, we tested thiamine blood levels in the context of IRS. IRS is a serious complication that can occur during oral refeeding of a malnourished patient. Hypophosphataemia is a hallmark feature and may be associated with hypomagnesaemia and hypocalcaemia, as well as thiamine deficiency [11].

It takes 4–6 weeks to deplete the body's thiamine reserves [12]. The long-term consequences of severe thiamine deficiency include haemorrhagic and/or necrotic skin lesions, loss of neurones and dendritic spines in the thalamus and hypothalamus, and polyneuritis [13]. Flaccid tetraparesis, predominantly in the lower limbs, was observed in the neurological examination of our patient. EMG of all four extremities revealed severe sensory axonal neuropathy predominantly affecting the nerves of the lower limbs. A nerve biopsy was not performed, but it would have confirmed axonal degeneration [14]. The management of this nerve damage requires long-term multidisciplinary follow-up. Recovery is slow in the presence of extensive axonal degeneration.

### 3.2. The Relationship between Thiamine Nutritional Status and Depressive Symptoms

Thiamine deficiency and depression can have overlapping symptoms. The initial symptoms of thiamine deficiency are non-specific and usually develop insidiously. They include loss of appetite, nausea, weakness, apathy, fatigue, irritability, sleep disturbances, and anorexia [15, 16]. In our patient, thiamine levels were significantly decreased in the early days of hospitalisation. Clinically, weakness, fatigue, and anorexia were also marked.

The relationship between thiamine and depression has garnered attention in research. Indeed, depression can induce alterations in appetite and food consumption. Consequently, it remains uncertain whether reduced vitamin B intake precedes the onset of depression or arises as a result of it. Several studies have shown an inverse association between depressive symptoms and thiamine levels in adults. In a cross-sectional study conducted in China, subjects with lower erythrocyte thiamine levels had more severe depression symptoms [17]. Additionally, a positive correlation between thiamine deficiency and depressive symptoms was found in a population of 74 individuals with malnutrition [18]. In our case, the improvement in thymic symptoms was remarkable with both antidepressant and thiamine supplementation. Ghaleiha et al. [19] studied the effect of thiamine supplementation in subjects deficient in thiamine and suffering from major depressive disorder. The study concluded that depressive symptoms significantly improved in subjects with major depressive disorder after 6 weeks of thiamine supplementation compared with placebo [19].

To date, the neurophysiological mechanisms underlying the effect of thiamine on mood are not fully understood. However, interest in brain thiamine homeostasis is rapidly expanding because abnormalities in thiamine-dependent enzymes, oxidative stress, and diminished metabolism occur in several neurodegenerative and mood disorders [20]. Thiamine diphosphate, the most bioactive form of thiamine, is crucial for nerve conduction as well as the biosynthesis and the secretion of serotonin [21, 22]. Ke and Gibson have reported that thiamine deficiency can lead to mitochondrial dysfunction and chronic oxidative stress. Furthermore, both mitochondrial dysfunction and chronic oxidative stress contribute to depression [17, 20].

There are several neurophysiological frameworks to explain the positive influence of thiamine on mood and depression, though future research might conduct further studies both on animals and on humans to gain more insight into the underlying neurophysiological mechanisms.

## 4. Conclusion

This case underscores the critical importance of recognising and addressing thiamine deficiency in individuals with severe depressive disorders, particularly those exhibiting melancholic symptoms and significant weight loss. Despite the well-established association between thiamine deficiency and malnutrition, routine screening for this essential vitamin is often overlooked in cases involving underlying psychiatric illnesses.

Emerging research has shed light on the role of thiamine in the etiopathogenesis of mood disorders. This highlights the significance of thiamine not only in preventing complications but also in understanding the root causes of mood disorders. Prompt detection and intervention can play a crucial role in averting prolonged disability and enhancing the overall quality of life for individuals grappling with severe depression and hypovitaminosis B1.

## Figures and Tables

**Table 1:** Results of electromyography showing absent amplitude response of the lower limbs sensory nerve and significant reduction of the upper limb amplitudes.

Nerve	Latency (ms)	Amplitude (*µ*v)	Speed (m/s)	Surface (*µ*V × ms)	Duration (ms)	Distance (mm)
Left median sensory						
Digit 2-wrist	3.94	9.7	35.5	15.2	3.9	140
Right superficial fibular sensory						
Leg–ankle	—	—	—	—	—	100
Left radial sensory						
Forearm-D1	3.46	3.5	28.9	1.39	2.1	100
Left sural sensory						
Leg–ankle	—	—	—	—	—	140
Right ulnar sensory						
D5-wrist	3.09	1.79	38.8	0.55	1.91	120

## Data Availability

Data generated or analysed during this study are included in this article. Further enquiries can be directed to the corresponding author.
